# Endoplasmic Reticulum Stress Is Involved in Stress-Induced Hypothalamic Neuronal Injury in Rats via the PERK-ATF4-CHOP and IRE1-ASK1-JNK Pathways

**DOI:** 10.3389/fncel.2019.00190

**Published:** 2019-05-03

**Authors:** Shanyong Yi, Ke Chen, Lihua Zhang, Weibo Shi, Yaxing Zhang, Shiba Niu, Miaomiao Jia, Bin Cong, Yingmin Li

**Affiliations:** Hebei Key Laboratory of Forensic Medicine, Collaborative Innovation Center of Forensic Medical Molecular Identification, Department of Forensic Medicine, Hebei Medical University, Shijiazhuang, China

**Keywords:** hypothalamus, stress, neuronal injury, PERK-ATF4-CHOP pathway, IRE1-ASK1-JNK pathway

## Abstract

**Objective:**

As a high-level nerve center that regulates visceral and endocrine activity, the hypothalamus plays an important role in regulating the body’s stress response. Previous studies have shown that stress can cause damage to hypothalamic neurons. The present study aimed to further clarify the mechanism of endoplasmic reticulum stress (ERS) involvement in hypothalamic neuronal injury.

**Methods:**

A 7-day stressed rat model was established with daily restraining for 8 h and forced ice-water swimming for 5 min. The rats were randomly divided into control, stress, stress + GSK2606414 (PERK phosphorylation inhibitor), stress + KIRA6 (IRE1 phosphokinase activity inhibitor), GSK2606414, and KIRA6 groups. The pathological changes of hypothalamic neurons were observed by thionine staining. Expression of ERS proteins GRP78, ATF4, ASK1, JNK, and CHOP in the hypothalamic neurons were observed by immunohistochemical staining. The expression of JNK and CHOP mRNA in the hypothalamic neurons were observed by RNA *in situ* hybridization (RNA Scope) and the expression of related proteins and mRNA was semiquantitatively analyzed by microscopy-based multicolor tissue cytometry (MMTC).

**Results:**

Thionine staining revealed that stress exposure resulted in edema, a lack of Nissl bodies, and pyknosis in hypothalamic neurons. Immunohistochemistry and RNA Scope showed that stress exposure significantly increased the expression of GRP78, ATF4, ASK1, CHOP, JNK, JNK mRNA, and CHOP mRNA. Treatment with PERK and IRE1 inhibitors attenuated pathological damage and downregulated the expression of ATF4, ASK1, JNK, CHOP, JNK mRNA, and CHOP mRNA.

**Conclusion:**

Stress caused pathological changes in rat hypothalamic neurons. ERS PERK-ATF4-CHOP and IRE1-ASK1-JNK pathways were involved in the injury process.

## Introduction

Stress is a systemic, non-specific reaction in response to external, or internal threatening stimuli (Reser, 2016). After a long period of evolution, organisms formed a complete and highly conservative regulatory system; this regulatory system is composed of the immune, cardiovascular and neuroendocrine systems, which can cope with internal or external stimuli and respond adaptively (Nicolaides et al., 2015; Fonken et al., 2018). Moderate stress causes the stress response, including changes in behavior and hormone levels, even in gene expression, morphology, and physiology for environmental adaptability (Sun and Zhou, 2018). When the stress intensity exceeds the adjustment capacity of the body, it can cause damage or death (Marin et al., 2011).

The hypothalamus-pituitary-adrenal (HPA) axis plays a critical role in the body’s stress response. The hypothalamus is activated when the body is stimulated by internal or external risk factors, which then promotes adrenal cortex synthesis, and the release of glucocorticoids through the HPA axis; Glucocorticoids can enhance the body’s resistance to risk factors by mobilizing energy and regulating metabolism, while long-term stress exposure or excessive stress can also lead to varying degrees of damage, and dysfunction (McLaughlin et al., 2007; Vegas et al., 2018). In a previous study, we observed that serum glucocorticoid levels significantly increased in stressed rats and stress led to pathological damage in the hypothalamus (Yi et al., 2017). However, there is no detailed report on the damage related to this mechanism.

As an important organelle involved in the synthesis, folding, modification and secretion of proteins under normal conditions, the protein folding ability of the endoplasmic reticulum matches with the body’s protein synthesis ability (Woehlbier and Hetz, 2011; Hetz and Saxena, 2017). Various types of internal and external stimuli can disturb cells through various signaling pathways, leading to an accumulation of unfolded or misfolded proteins in the endoplasmic reticulum, causing endoplasmic reticulum stress (ERS); simultaneously, the body’s adaptation mechanism is activated to cope with unfolded or misfolded protein accumulation, i.e., the unfolded protein reaction (UPR) (Sokka et al., 2007). PERK and IRE1 are important sensory elements and major injury pathway initiation factors of ERS; when ERS occurs, they are separated from GRP78 to activate downstream signaling pathways, then restore ER homeostasis by reducing protein translation and promoting chaperone production (Bergmann and Molinari, 2018). Conversely, when the body is under severe or long-term ERS, ATF4 and ASK1 are, respectively activated by the PERK and IRE1 signaling pathways to promote the upregulation of CHOP and JNK. The sustained expressions of CHOP and JNK can induce cell death (Kim et al., 2017; Morris et al., 2018).

However, it remains unclear whether the PERK-ATF4-CHOP and IRE1-ASK1-JNK signaling pathways are associated with the injured hypothalamic neurons after stress exposure. For the present study, we successfully established a rat model with restraint and ice water swimming stress by simulating modern psychosocial and physiological stress. Then, we investigated pathological changes of hypothalamic neurons, ERS-related proteins, and mRNA changes to explore whether ERS is involved in the hypothalamic neuron injury process in stressed rats and its detailed mechanism.

## Materials and Methods

### Experimental Animals

Adult male Sprague-Dawley (SD) rats (Experimental Animal Center, Hebei Medical University, China), weighing 250 ± 20 g, had *ad libitum* access to food and water in a room with an ambient temperature of 22± 2°C and a 12:12-h light/dark cycle. This study was approved by the Institutional Review Board for Animal Experiments at Hebei Medical University. Every attempt was made to reduce the number of animals and to minimize pain and suffering. The rats were randomly divided into the following groups: control, 7 days of restraint stress combined with ice water swimming (stress), stress+PERK inhibitor GSK2606414 (stress+GSK2606414), stress+IRE1 inhibitor KIRA6 (stress+KIRA6), GSK2606414, and KIRA6 (*n* = 6 rats per group).

### Animal Treatments

For the stress+GSK2606414 and GSK2606414 groups, rats were fed GSK2606414 (Millipore, 516535, Burlington, MA, United States) by oral gavage (in vehicle: 0.5% HPMC, 0.1% Tween-80 in water, pH 4.0) at a dose of 10 mg/kg once a day for 7 days. For the stress+ KIRA6 and KIRA6 groups, rats were i.p., injected with KIRA6 (Millipore, 532281, Burlington, MA, United States; in vehicle: 3% ethanol, 7% Tween-80, 90% saline) at a dose of 10 mg/kg once a day for 7 days. Then, the rats requiring restraint treatment were placed in the restrainer with no access to food and water for 8 h (from 8:00 to 16:00) each day. The stress protocol was adapted from a previously described method (Imbe et al., 2012); the rats could stretch their legs, but could not move within the restrainers. Then, the restricted rats were placed in ice water to swim for 5 min each day. The stress-inducing exercises lasted for 7 days. The control, GSK2606414, and KIRA6 groups rats were left in the cages for the same time without food and water. During the rest period, all rats were provided food and water *ad libitum*.

### Tissue Preparation

**FIGURE 1:**
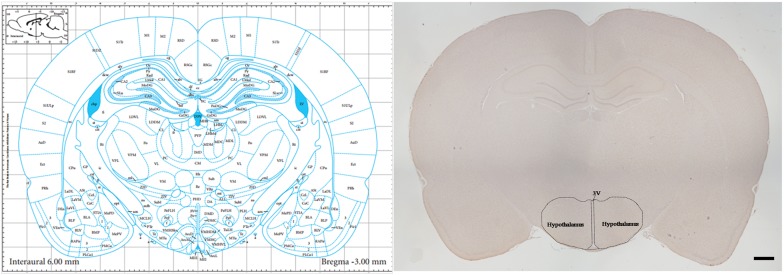
The section* with the largest area of the Hypothalamus.

Tissue Preparation was performed as described previously (Yi et al., 2017). Tissue used for staining was harvested and fixed immediately in 10% formalin. The tissue was subsequently dehydrated in a graded ethanol series and embedded in paraffin. Brain slices beginning at –1.80 mm from bregma were obtained using a stereotaxic atlas (Paxinos and Watson, 2007) and a rotary microtome (Leica RM2255, Shanghai, China). For hypothalamus analysis, consecutive 5-μm-thick coronal sections with the largest hypothalamus area that corresponded to -3.0 mm from bregma were collected, as per the Paxinos and Watson atlas (Figure 1). Sections were prepared for thionine staining, mRNA *in situ* hybridization (RNAscope) and immunohistochemical staining and examined under a light microscope (Olympus IX71; Olympus, Tokyo, Japan).

### Thionine Staining

Deparaffinized sections were stained with 4% thionine for 90 s at a temperature of 60°C, then dehydrated by graded alcohol and mounted with neutral gum.

### Immunohistochemistry

Immunohistochemistry was performed as described previously (Yi et al., 2017) Deparaffinized sections were pretreated using microwave antigen retrieval, followed by incubation in 3% H_2_O_2_ in cold methanol for 30 min and goat serum for 30 min. Next, the tissues were incubated overnight at 4°C with antibodies against GRP78 (Cat.No. ab188878, 1:100, Abcam, Cambridge, MA, United States), ATF4 (Cat.No. ab186297, 1:100, Abcam, Cambridge, MA, United States), ASK1 (Cat.No. A3271, 1:200, ABclonal, Wuhan, Hubei, China), JNK (Cat.No. A11119, 1:200, ABclonal, Wuhan, Hubei, China), and CHOP (Cat.No. ab 179823, 1:100, Abcam, Cambridge, MA, United States). The tissues were then incubated for 1 h with biotinylated secondary antibody and subsequently with horseradish peroxidase (HRP)-conjugated biotin for 30 min. Finally, 3, 3′-diaminobenzidine (DAB) was used as the chromagen. The tissues were counterstained with hematoxylin to visualize locations in the sections. The primary antibodies were replaced by 0.01 mmol/L PBS in the negative controls (not shown).

### mRNA *in situ* Hybridization (RNAscope)

The samples were analyzed with an RNAScope assay (Advanced Cell Diagnostics, lnc, Hayward, CA, United States) using the RNAscope 2.5 HD Reagent-Red kit (LOT: 2002384) and the RNAscope H_2_O_2_ & Protease Plus Reagents kit (LOT: 2003020). The procedure was performed following the manufacturer’s instructions. Deparaffinized sections were dried for 1 h at 60°C, deparaffinized with xylene and 100% ethanol, incubated with the hydrogen peroxide solution for 10 min at room temperature, followed by incubation in target retrieval reagents solution for 15 min at 99°C and protease solution for 30 min at 40°C. The sections were incubated with targeted probes: Rn-CHOP (LOT: 18075A), Rn-JNK (LOT: 18310B), positive control probe-Rn-PPIB (LOT: 18164A), and negative control probe-dapb (LOT: 18240A). The hybridization procedure was performed for 2 h at 40°C. Then, the sections were incubated with the signal amplification solution HD 2.5 detection kit: amp1 for 30 min at 40°C, amp2 for 15 min at 40°C, amp3 for 30 min at 40°C, amp4 for 15 min at 40°C, amp5 for 30 min at room temperature, amp6 for 15 min at room temperature, and finally with a mixture of Fast-RED solutions A and B (1:60) for 10 min at room temperature. The tissues were counterstained with hematoxylin to visualize locations in the sections.

### Cell Counting

Cell counting was performed as described previously (Yi et al., 2017). Six rats from each group were used for morphological observation and data analysis. According to the stereotaxic atlas (Paxinos and Watson, 2007), the largest hypothalamus area was accurately exposed. Using the serial section technique, we took one out of every five sections and selected a total of three sections for each rat. With microscopy based multicolor tissue cytometry (MMTC, TissueGnostics, Beijing, China), we evaluated the percentage of positive cells in the whole hypothalamic area of each section. The data of each rat was derived from the average of those three sections. MMTC has been used by previous researchers (Ecker et al., 2006) and has the advantage of being more objective than subjective assessment by an investigator. Sections were analyzed at 100× field view using a Tissuefax Plus system based on the ZeissR AxioImagerZ2 Microscope (Jena, Germany). Images were acquired with the TissueFaxs (Tissue-Gnostics R, Vienna, Austria) software. The percentage of GRP78-, ATF4-, ASK1-, JNK-, and CHOP-positive cells in the largest hypothalamus area was quantified using HistoQuest R (TissueGnostics) software. HistoQuest R is an analytical tool used to quantify immunostaining based on single cells using the cell-specific nucleus structure as the primary identification marker (hematoxylin), followed by an automatic segmentation of the immunostaining confined to the corresponding nucleus. A ring mask around this nucleus is interactively defined and set as the parameter for sections stained with a certain marker-specific channel called the single reference shade. The brown staining caused by chromogen (3,3’- diaminobenzidine, DAB) is automatically separated from the blue hematoxylin staining into their optical density counterparts. The mean optical density per cell is quantified by the segmentation method. The region of interest was defined for the largest hypothalamus area. Identification of neurons was accomplished through morphometric parameters such as the nuclear size and shape. A background threshold for hematoxylin staining was determined interactively. Immunostaining cutoffs were determined as well (this tool differentiates between positive and negative cells; these were set in the dot blots). All images were acquired with the same parameters. The representative brown color (DAB chromogen) was selected by the color picker tool. Positive staining cells in the largest hypothalamus area were shown in the scattergram of forward gating tool. The raw data of the analysis were imported into SPSS 21.0 (IBM, Armonk, NY, United States) for further statistical analysis. The number of GRP78-, ATF4-, ASK1-, JNK-, and CHOP-positive immunostaining cells was divided by the total number of hypothalamic neurons, yielding a percentage of positive cells.

For the RNAScope experiment, the above methods was also used to count the number of neurons and the number of RNAScope positive points (RED chromogen) in the whole hypothalamic region, then the average number of CHOP and JNK mRNA positive points per neuron was obtained.

### Statistical Analysis

Using the method of Kolmogorov-Smirnov Test, the data was normal distribution among all groups (*P* > 0.1). The results are presented as the mean ± SEM. Due to the normal distribution in all samples, statistical analysis was performed using a one-way ANOVA. *Post hoc* LSD *t*-tests were used when comparisons were restricted to two experimental groups. The threshold for statistical significance was defined as *P* < 0.05, value of *P* < 0.01 is considered as significant difference.

## Results

### Thionine Staining Shows Pathological Changes in Hypothalamic Neurons

**FIGURE 2:**
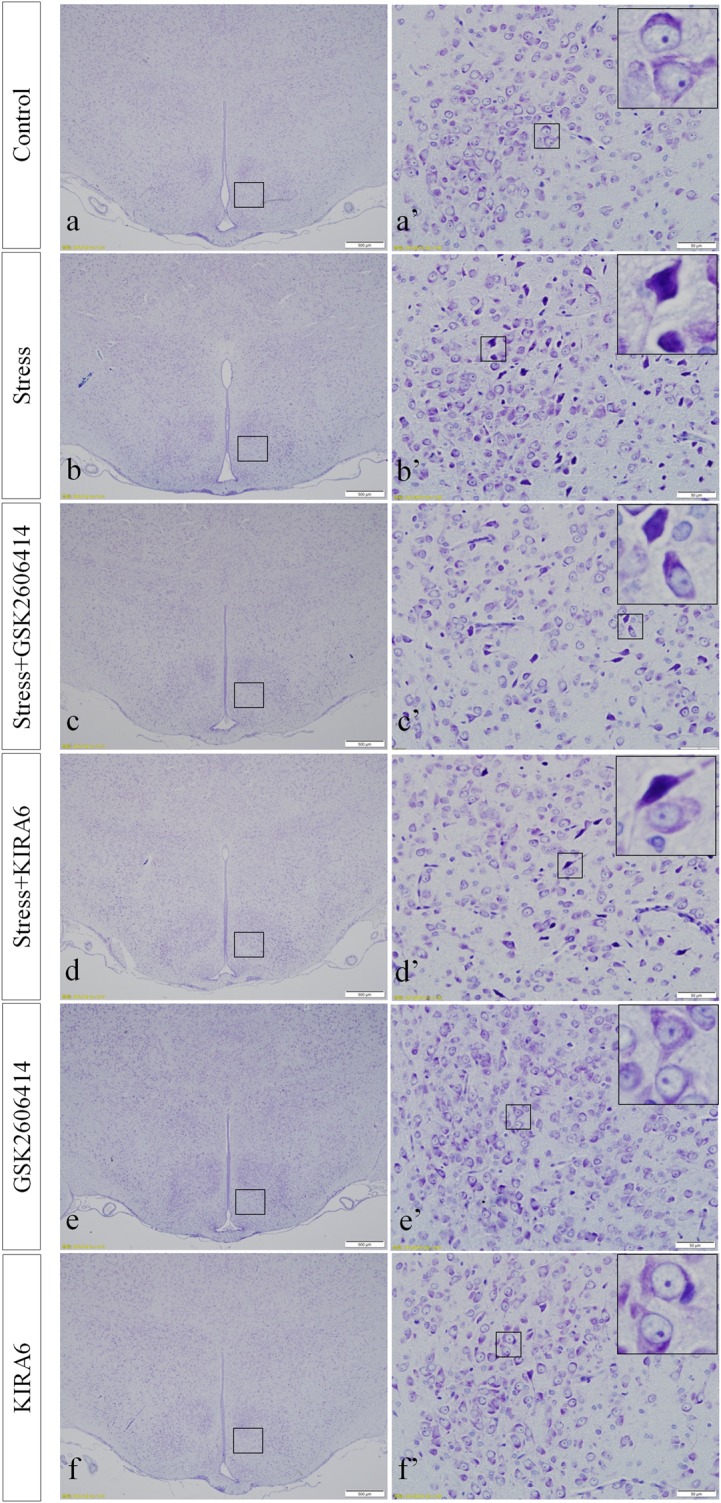
Thionine staining of the hypothalamus. **(a’**–**f’)** are magnified areas of **(a**–**f)**, respectively. High-power photomicrographs in the right corners of **(a’**–**f’)** are enlarged from rectangles in the low-power photomicrographs. Bars = 500 μm in **(a**–**f)**; Bars = 50 μm in **(a’**–**f’)**.

In the control group, the neuronal structures were clear, and Nissl bodies were evenly distributed in the cytoplasm. Compared with the control group, there were no significant changes in Nissl bodies in the GSK2606414 and KIRA6 groups; after 7 days of stress stimulation, some Nissl bodies disappeared and pyknotic neurons were visible; associated damage significantly moderated in the stress+GSK2606414 and stress+KIRA6 groups (Figure 2).

### GRP78, ATF4, ASK1, JNK, and CHOP Protein Expression in the Hypothalamus

GRP78, ATF4, ASK1, JNK, and CHOP proteins are located in the cytoplasm and were stained brown by immunohistochemical staining in the present experiment.

**FIGURE 3:**
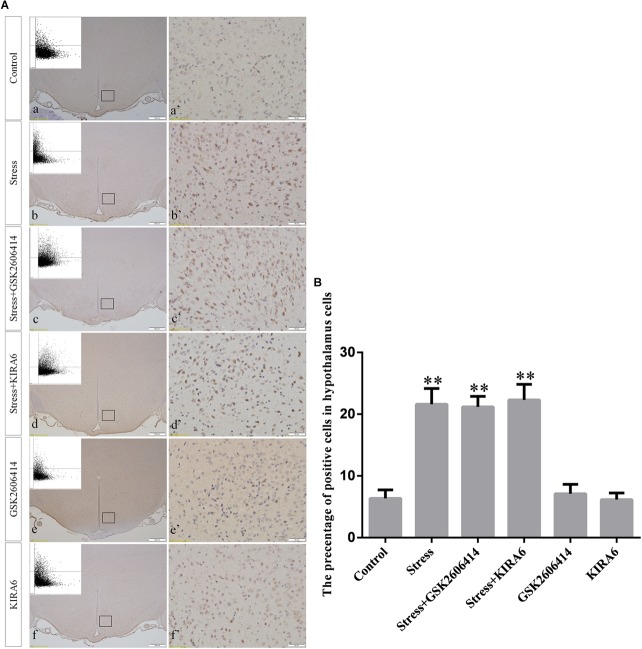
**(A)** Representative images showing GRP78 immunohistochemistry in the hypothalamus. **(a’**–**f’)** are magnified areas of **(a**–**f)**, respectively. Representative images obtained by microscopy-based multicolor tissue cytometry (MMTC) are shown in the left corners of **(a**–**f)**. Bars = 500 μm in **(a**–**f)**; Bars = 50 μm in **(a’**–**f’)**. **(B)** Quantitative MMTC analysis. The data are shown as the mean ± SEM, ^∗∗^*P* < 0.01 vs. control group (*n* = 6).

**FIGURE 4:**
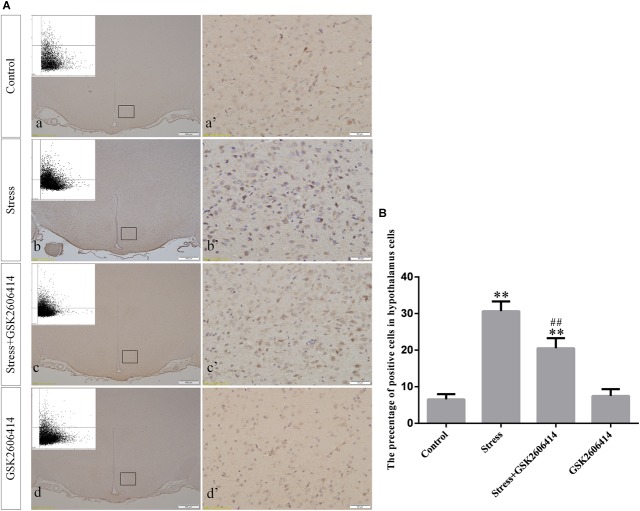
**(A)** Representative images showing ATF4 immunohistochemistry in the hypothalamus. **(a’**–**d’)** are magnified areas of **(a**–**d)**, respectively. Representative images obtained by MMTC are shown in the left corners of **(a**–**d)**. Bars = 500 μm in **(a**–**d)**; Bars = 50 μm in **(a’**–**d’)**. **(B)** Quantitative MMTC analysis. The data are shown as the mean ± SEM, ^∗∗^*P* < 0.01 vs. control group; ^##^*P* < 0.01 vs. stress group (*n* = 6).

**FIGURE 5:**
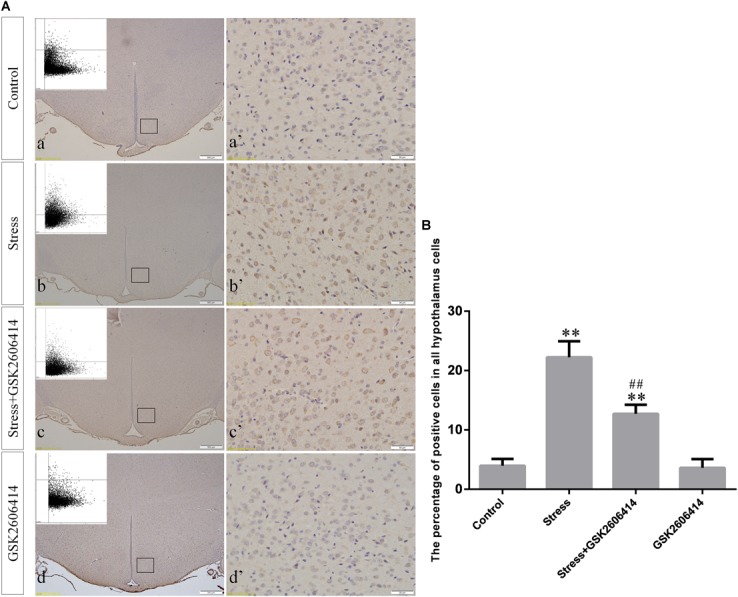
**(A)** Representative images showing CHOP immunohistochemistry in the hypothalamus. **(a’**–**d’)** are magnified areas of **(a–d)**, respectively. Representative images obtained by MMTC are shown in the left corners of **(a**–**d)**. Bars = 500 μm in **(a**–**d)**; Bars = 50 μm in **(a’**–**d’)**. **(B)** Quantitative MMTC analysis. The data are shown as the mean ± SEM, ^∗∗^*P* < 0.01 vs. control group; ^##^*P* < 0.01 vs. stress group (*n* = 6).

**FIGURE 6:**
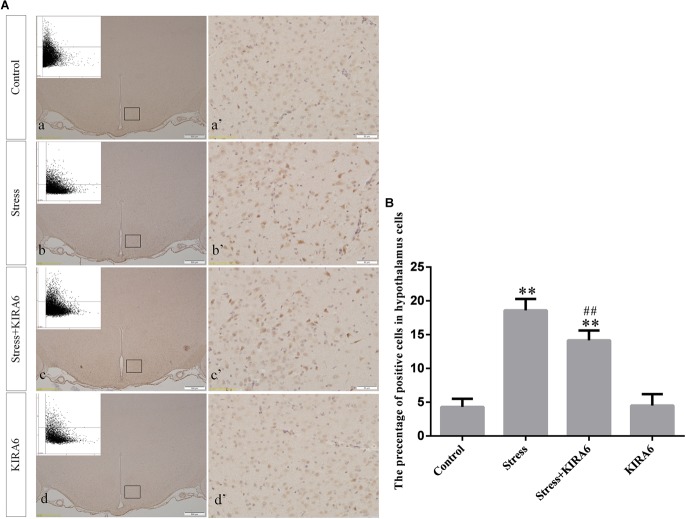
**(A)** Representative images showing ASK1 immunohistochemistry in the hypothalamus. **(a’**–**d’)** are magnified areas of **(a**–**d)**, respectively. Representative images obtained by MMTC are shown in the left corners of **(a**–**d)**. Bars = 500 μm in **(a**–**d)**; Bars = 50 μm in **(a’**–**d’)**. **(B)** Quantitative MMTC analysis. The data are shown as the mean ± SEM, ^∗∗^*P* < 0.01 vs. control group; ^##^*P* < 0.01 vs. stress group (*n* = 6).

**FIGURE 7:**
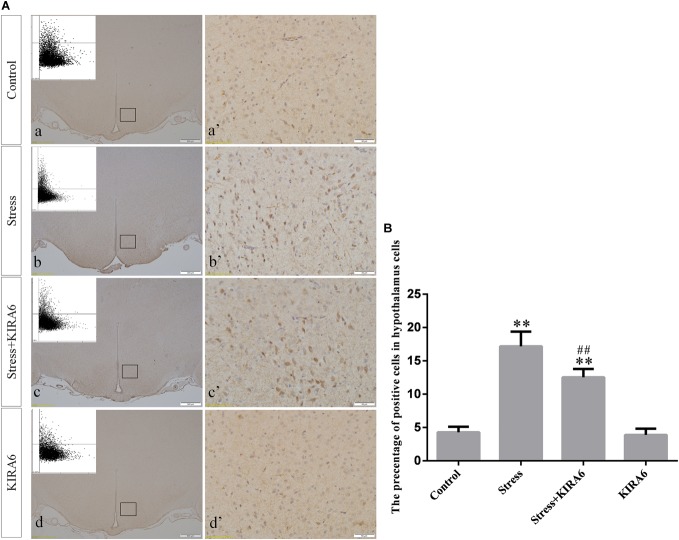
**(A)** Representative images showing JNK immunohistochemistry in the hypothalamus. **(a’**–**d’)** are magnified areas of **(a**–**d)**, respectively. Representative images obtained by MMTC are shown in the left corners of **(a–d)**. Bars = 500 μm in **(a–d)**; Bars = 50 μm in **(a’–d’)**. **(B)** Quantitative MMTC analysis. The data are shown as the mean ± SEM, ^∗∗^*P* < 0.01 vs. control group; ^##^*P* < 0.01 vs. stress group (*n* = 6).

Compared with the control group (6.37 ± 0.56), GRP78 expression remained at a low level in the GSK2606414 (7.11 ± 0.62, *P* > 0.05) and KIRA6 (6.16 ± 0.45, *P* > 0.05) groups and was significantly upregulated in the stress (21.62 ± 1.04, *P* < 0.01), stress+GSK2606414 (21.19 ± 0.69, *P* < 0.01) and stress+KIRA6 (22.32 ± 1.03, *P* < 0.01) groups (Figure 3). Compared with the control group (6.57 ± 0.57), ATF4 expression remained at a low level in the GSK2606414 group (7.50 ± 0.76, *P* > 0.05) and significantly increased in the stress (30.66 ± 1.08, *P* < 0.01) and stress+GSK2606414 (20.50 ± 1.13, *P* < 0.01) groups; compared with the stress group, ATF4 expression significantly decreased in the stress+GSK2606414 (*P* < 0.01) group (Figure 4). Compared with the control group (3.96 ± 0.47), CHOP expression remained at a low level in the GSK2606414 group (3.62 ± 0.60, *P* > 0.05) and significantly increased in the stress (22.23 ± 1.10, *P* < 0.01) and stress+GSK2606414 (12.71 ± 0.62, *P* < 0.01) groups; compared with the stress group, CHOP expression significantly decreased in the stress+GSK2606414 (*P* < 0.01) group (Figure 5). Compared with the control group (4.30 ± 0.49), ASK1 expression remained at a low level in the KIRA6 group (4.51 ± 0.69, *P* > 0.05) and significantly increased in the stress (18.59 ± 0.59, *P* < 0.01) and stress+KIRA6 (14.15 ± 0.60, *P* < 0.01) groups; compared with the stress group, ASK1 expression significantly decreased in the stress+KIRA6 group (*P* < 0.01) (Figure 6). Compared with the control group (4.28 ± 0.34), JNK expression remained at a low level in the KIRA6 group (3.88 ± 0.38, *P* > 0.05) and significantly increased in the stress (17.18 ± 0.90, *P* < 0.01) and stress+KIRA6 (12.53 ± 0.51, *P* < 0.01) groups; compared with the stress group, JNK expression significantly decreased in the stress+KIRA6 group (*P* < 0.01) (Figure 7).

### CHOP and JNK mRNA Expression in the Hypothalamus

**FIGURE 8:**
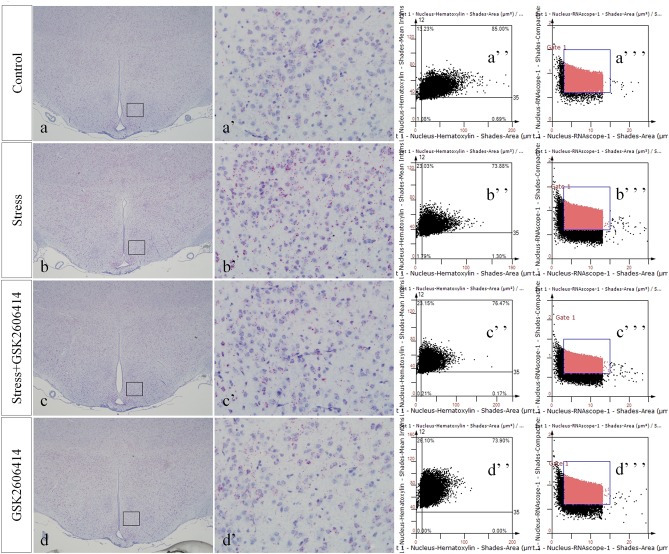
Representative images showing CHOP RNAScope in the hypothalamus. **(a’**–**d’)** are magnified areas of **(a**–**d)**, respectively. **(a”**–**d”)** and **(a”’**–**d”’)** are representative images obtained by MMTC. **(a”**–**d”)** show the number of neurons and **(a”’**–**d”’)** show the number of RNAScope positive points in the hypothalamus. Bars = 500 μm in **(a–d)**; Bars = 50 μm in **(a’**–**d’)**.

**FIGURE 9:**
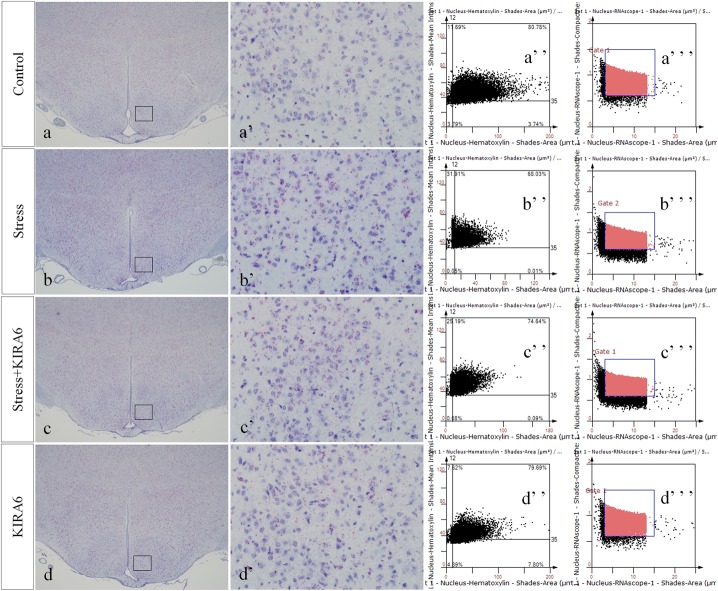
Representative images showing JNK RNAScope in the hypothalamus. **(a’**–**d’)** are magnified areas of **(a**–**d)**, respectively. **(a”**–**d”)** and **(a”’**–**d”’)** are representative images obtained by MMTC. **(a”**–**d”)** show the number of neurons and **(a”’**–**d”’)** show the number of RNAScope positive points in the hypothalamus. Bars = 500 μm in **(a**–**d)**; Bars = 50 μm in **(a’**–**d’)**.

**FIGURE 10:**
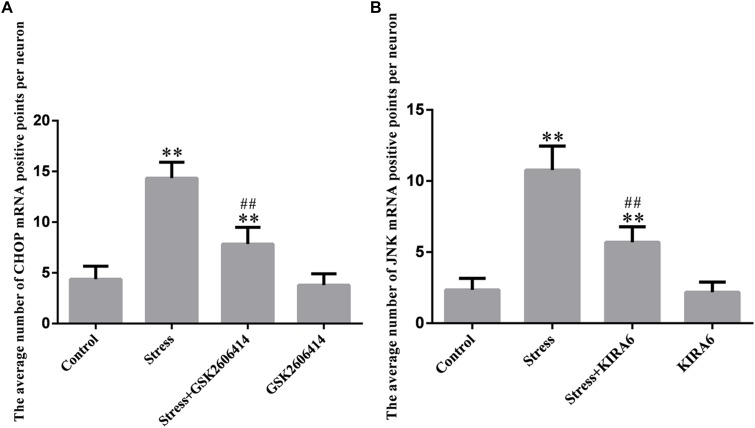
Quantitative MMTC analysis. The data are shown as the mean ± SEM, ^∗∗^P < 0.01 vs. control group; ^##^P < 0.01 vs. stress group (*n* = 6).

Compared with the control group (4.38 ± 0.52), CHOP mRNA expression remained at a low level in the GSK2606414 group (3.80 ± 0.46, *P* > 0.05) and significantly increased in the stress (14.34 ± 0.64, *P* < 0.01) and stress+GSK2606414 (7.84 ± 0.68, *P* < 0.01) groups; compared with the stress group, CHOP mRNA expression significantly decreased in the stress+GSK2606414 group (*P* < 0.01) (Figure 8, 10A). Compared with the control group (2.34 ± 0.33), JNK mRNA expression remained at a low level in the KIRA6 group (2.18 ± 0.30, *P* > 0.05) and significantly increased in the stress (10.77 ± 0.69, *P* < 0.01) and stress+KIRA6 (5.70 ± 0.44, *P < 0.01*) groups; compared with the stress group, JNK mRNA expression significantly decreased in the stress+KIRA6 group (*P* < 0.01) (Figure 9, 10B).

## Discussion

In the rapidly developing modern society, stress is an inevitable life experience. Moderate stress, such as physical exercise and good social activity, have beneficial effects on our body, but when stress is too severe and/or lasts too long, it can lead to a series of injuries to the body, such as nervous, cardiovascular, endocrine, and autoimmune diseases (Chrousos, 2009; Marin et al., 2011; Hetz, 2012). The present study constructed a rat model with restraint and ice-water swimming stress, aiming to simulate the impact of complex psychological, and physiological stress on the structure and function of the body to better understand the damage mechanism of stress to the body and provide countermeasures.

The hypothalamus can be activated by various internal and external stimulating factors via a variety of signaling pathways, promoting the adrenal cortex to synthesize, and release glucocorticoids through the HPA axis; this process can establish good body adaptability (Kadmiel et al., 2016). It has also been reported that stress can cause dysfunction of the HPA axis, leading to negative effects (Yi et al., 2017; Starr et al., 2019). In the present experiment, the result of thionine staining revealed that stress exposure led to reduced Nissl bodies in hypothalamic and pyknotic neurons. Pathological changes in morphology must be accompanied by functional changes, which may affect the function of the HPA axis and reduce the body’s resistance to stress.

Endoplasmic reticulum stress is an important step in the progression of bodily damage. Current research has confirmed that ERS not only participates in the occurrence and progression of various neurodegenerative diseases, including Parkinson’s and Alzheimer disease (Kim et al., 2005; Hoozemans et al., 2012), but also participates in the pathological process of dysfunction caused by ischemia and hypoxia (Tajiri et al., 2004; Sanderson et al., 2015; Yang and Hu, 2015). When ERS occurs, it triggers a series of cellular reactions, such as decreased protein synthesis and increased endoplasmic reticulum (ER) chaperone synthesis (Ron, 2002; Rutkowski and Kaufman, 2007). As a marker of ERS, the molecular chaperone GRP78 can bind to the hydrophobic residue of misfolded or unfolded protein, reduce the accumulation of misfolded or unfolded proteins in the endoplasmic reticulum, and restore the physiological function of the endoplasmic reticulum (Lee, 2005). In addition, GRP78 can bind to the pro-apoptotic receptors activated by ERS, and suppress its downstream signaling pathway, thereby protecting cells and maintaining homeostasis (Grkovic et al., 2013). In the present study, the expression level of GRP78 in the stress group was significantly higher than that in the control group, suggesting that ERS occurred in the hypothalamus of stressed rats, and the mechanism of stress protection was initiated.

However, when stress stimuli persist, ERS signaling pathways related to cellular damage are activated, leading to cell damage and even apoptosis (Sanderson et al., 2015). This may be related to the abovementioned pathological changes such as neuronal pyknosis and disappearance of Nissl bodies.

When ERS occurs, PERK can form oligomers by autophosphorylation, which promote the phosphorylation of its downstream eukaryotic translation initiation factor 2α (eIF2α), thereby reducing cell stress and allowing cells to survive by inhibiting protein synthesis (Schroder and Kaufman, 2005). However, excessive or persistent ERS can skip the eIF2α phosphorylation and activate activating transcription factor 4 (ATF4), the downstream transcription factor of PERK (Wu et al., 2018). Activated ATF4 can increase the transcription and expression level of CHOP. It is well known that CHOP is a key pro-apoptotic molecule that can lead to bodily damage by arresting the cell cycle and inducing cell death (Oyadomari and Mori, 2004; Tajiri et al., 2004). The results of the present study showed that stress caused a significant increase in the expression of ATF4, CHOP, and CHOP mRNA. GSK2606414 can specifically inhibit the autophosphorylation of PERK, thereby preventing the activation of downstream ATF4-CHOP signaling pathway. After the application of PERK phosphorylation inhibitor GSK2606414, the expression of ATF4, CHOP, and CHOP mRNA were significantly decreased, and the results of thionine staining showed that the degree of cell damage was significantly reduced, suggesting that the PERK-ATF4-CHOP pathway is involved in stress-induced hypothalamic neuronal injury.

IRE1 has two isoforms that exist in different parts: IRE1α and IRE1β. The former is widely distributed in mammalian cells, and the latter is only expressed in intestinal epithelial cells. Under normal conditions, similarly to PERK, IRE1 combines with the molecular chaperone to form a stable complex. When ERS occurs, IRE1α is separated from the chaperone, then autophosphorylated to activate its endonuclease activity (Gardner and Walter, 2011), which can cleave XPB1mRNA to generate active sXPB1mRNA, which promotes the expression of ER and molecular chaperone P58IPK. P58IPK can remove the inhibition of protein translation by binding to PERK, solving the mild unfolded protein overload (Yan et al., 2002; Zhou et al., 2011). However, excessive or sustained ERS can also activate the phosphokinase activity of IRE1, sensitizing the downstream apoptotic signal-regulated kinase 1. Activated ASK1 induces and increases JNK expression by increasing its transcription (Szegezdi et al., 2006). The results of the present study showed that stress caused a significant increase of the expression of ASK1, JNK, and JNK mRNA. KIRA6 can specifically inhibit the phosphokinase activity of IRE1, thereby preventing the activation of downstream ASK1-JNK signaling pathway. After the application of IRE1 phosphokinase inhibitor KIRA6, the expression of ASK1, JNK, and JNK mRNA were significantly decreased, and the results of thionine staining showed that the degree of cell damage was significantly reduced, suggesting that the IRE1-ASK1-JNK pathway is involved in stress-induced hypothalamic neuronal injury.

## Conclusion

In conclusion, the present study demonstrated that the PERK-ATF4-CHOP and IRE1-ASK1-JNK pathways are involved in the injury process of hypothalamic neurons in stressed rats, and these novel findings provide a pathomorphological basis for the mechanism of stress-induced hypothalamic neuronal injury.

## Ethics Statement

This study was approved by the Institutional Review Board for Animal Experiments at Hebei Medical University. Every attempt was made to reduce the number of animals and to minimize pain and suffering.

## Author Contributions

All authors wrote the manuscript, designed and performed the experiments, read, and commented on the manuscript. SY, KC, and LZ carried out the statistical analyses and organized the data. WS, YZ, and SN created the figures. MJ, YL, and BC supervised the research design and revised the manuscript.

## Conflict of Interest Statement

The authors declare that the research was conducted in the absence of any commercial or financial relationships that could be construed as a potential conflict of interest.

## References

[B1] Bergmann T. J. Molinari M. (2018). Three branches to rule them all? UPR signalling in response to chemically versus misfolded proteins-induced ER stress. *Biol. Cell.* 110 197–204. 10.1111/boc.201800029 29979817

[B2] Chrousos G. P. (2009). Stress and disorders of the stress system. *Nat. Rev. Endocrinol.* 5 374–381. 10.1038/nrendo.2009.106 19488073

[B3] Ecker R. C. Rogojanu R. Streit M. Streit M. Oesterreicher K. Steiner G. E. (2006). An improved method for discrimination of cell populations in tissue sections using microscopy-based multicolor tissue cytometry. *Cytometry* 69 119–123. 10.1002/cyto.a.20219 16479616

[B4] Fonken L. K. Matthew G. F. Andrew D. G. Steven F. M. (2018). Stress and aging act through common mechanisms to elicit neuroinflammatory priming. *Brain Behav. Immun.* 73 133–148. 10.1016/j.bbi.2018.07.012 30009999 PMC6129421

[B5] Gardner B. M. Walter P. (2011). Unfolded proteins are Ire1-activating ligands that directly induce the unfolded protein response. *Science* 333 1891–1894. 10.1126/science.1209126 21852455 PMC3202989

[B6] Grkovic S. Reilly V. C. Han S. Han S. Baxter R. C. Firth S. M. (2013). IGFBP-3 binds GRP78, stimulates autophagy and promotes the surrival of breast cancer cells exposed to adverse microenvironments. *Oncogene* 32 2412–2420. 10.1038/onc.2012.264 22751133

[B7] Hetz C. (2012). The unfolded protein response: controlling cell fate decisions under ER stress and beyond. *Nat. Rev. Mol. Cell Biol.* 13 89–102. 10.1038/nrm3270 22251901

[B8] Hetz C. Saxena S. (2017). ER stress and the unfolded protein response in neurodegeneration. *Nat. Rev. Neurol.* 13 477–491. 10.1038/nrneurol.2017.99 28731040

[B9] Hoozemans J. J. van Haastert E. S. Nijholt D. A. Rozemuller A. J. Scheper W. (2012). Activation of the unfolded protein response is an early event in Alzheimer’s and Parkinson’s disease. *Neurodegener. Dis.* 10 212–215. 10.1159/000334536 22302012

[B10] Imbe H. Kimura A. Donishi T. Kaneoke Y. (2012). Chronic restraint stress decreases glial fibrillary acidic protein and glutamate transporter in the periaqueductal gray matter. *Neuroscience* 223 209–218. 10.1016/j.neuroscience.2012.08.007 22890077

[B11] Kadmiel M. Janoshazi A. Xu X. Cidlowski J. A. (2016). Glucocorticoid action in human corneal epithelial cells establishes roles for corticosteroids in wound healing and barrier function of the eye. *Exp. Eye Res.* 152 10–33. 10.1016/j.exer.2016.08.020 27600171 PMC5097880

[B12] Kim A. J. Shi Y. Austin R. C. Werstuck G. H. (2005). Valproate protects cells from ER stress-induced lipid accumulation and apoptosis by inhibiting glycogen synthase kinase-3. *J. Cell Sci.* 118 89–99. 10.1242/jcs.01562 15585578

[B13] Kim J. Song H. Heo H. R. Kim J. W. Kim H. R. Hong Y. (2017). Cadmium-induced ER stress and inflammation are mediated through C/EBP-DDIT3 signaling in human bronchial epithelial cells. *Exp. Mol. Med.* 49:e372. 10.1038/emm.2017.125 28860664 PMC5628270

[B14] Lee A. S. (2005). The ER chaperone and signaling regulator GRP78/BiP as a monitor of endoplasmic reticulum stress. *Methods* 35 373–381. 10.1016/j.ymeth.2004.10.010 15804610

[B15] Marin M. F. Lord C. Andrews J. Juster R. P. Sindi S. Arsenault-Lapierre G. (2011). Chronic stress, cognitive functioning and mental health. *Neurobiol. Learn. Mem.* 96 583–595. 21376129 10.1016/j.nlm.2011.02.016

[B16] McLaughlin K. J. Gomez J. L. Baran S. E. Conrad C. D. (2007). The effects of chronic stress on hippocampal morphology and function: an evaluation of chronic restraint paradigms. *Brain Res.* 1161 56–64. 10.1016/j.brainres.2007.05.042 17603026 PMC2667378

[B17] Morris G. Puri B. K. Walder K. Berk M. Stubbs B. Maes M. (2018). The endoplasmic reticulum stress response in neuroprogressive diseases: emerging pathophysiological role and translational implications. *Mol. Neurobiol.* 55 8765–8787. 10.1007/s12035-018-1028-6 29594942 PMC6208857

[B18] Nicolaides C. N. Elli K. Agaristi L. George P. C. Evangelia C. (2015). Stress, the stress system and the role of glucocorticoids. *Neuroimmunomodulation* 22 6–19. 10.1159/000362736 25227402

[B19] Oyadomari S. Mori M. (2004). Roles of CHOP/GADD153 in endoplasmic reticulum stress. *Cell Death. Differ.* 11 381–389. 10.1038/sj.cdd.4401373 14685163

[B20] Paxinos G. Watson C. (2007). *The Rat Brain in Stereotaxic Coordinates*, 6th Edn. Amsterdam: Academic Press.

[B21] Reser J. E. (2016). Chronic stress, cortical plasticity and neuroecology. *Behav. process.* 129 105–115. 10.1016/j.beproc.2016.06.010 27334119

[B22] Ron D. (2002). Translational control in the endoplasmic reticulum stress response. *J. Clin. Invest.* 110 1383–1388. 10.1172/jci200216784 12438433 PMC151821

[B23] Rutkowski D. T. Kaufman R. J. (2007). That which does not kill me makes me stronger: adapting to chronic ER stress. *Trends Biochem. Sci.* 32 469–476. 10.1016/j.tibs.2007.09.003 17920280

[B24] Sanderson T. H. Gallaway M. Kumar R. (2015). Unfolding the unfolded protein response: unique insights into brain ischemia. *Int. J. Mol. Sci.* 16 7133–7142. 10.3390/ijms16047133 25830481 PMC4425008

[B25] Schroder M. Kaufman R. J. (2005). The mammalian unfolded protein response. *Annu. Rev. Biochem.* 74 739–789. 15952902 10.1146/annurev.biochem.73.011303.074134

[B26] Sokka A. L. Putkonen N. Mudo G. Pryazhnikov E. Reijonen S. Khiroug L. (2007). Endoplasmic reticulum stress inhibition protects against excitotoxic neuronal injury in the rat brain. *J Neurosci.* 27 901–908. 10.1523/jneurosci.4289-06.2007 17251432 PMC6672923

[B27] Starr L. R. Dienes K. Li Y. I. Shaw Z. A. (2019). Chronic stress exposure, diurnal cortisol slope, and implications for mood and fatigue: moderation by multilocus HPA-Axis genetic variation. *Psychoneuroendocrinology* 100 156–163. 10.1016/j.psyneuen.2018.10.003 30340064

[B28] Sun S. Zhou J. (2018). Molecular mechanisms underlying stress response and adaptation. *Thorac. Cancer* 9 218–227. 10.1111/1759-7714.12579 29278299 PMC5792716

[B29] Szegezdi E. Logue S. E. Gorman A. M. Samali A. (2006). Mediators of endoplasmic reticulum stress-induced apoptosis. *EMBO. Rep.* 7 880–885. 10.1038/sj.embor.7400779 16953201 PMC1559676

[B30] Tajiri S. Oyadomari S. Yano S. Morioka M. Gotoh T. Hamada J. I. (2004). Ischemia-induced neuronal cell death is mediated by the endoplasmic reticulum stress pathway involving CHOP. *Cell Death Differ.* 11 403–415. 10.1038/sj.cdd.4401365 14752508

[B31] Vegas O. Poligone B. Blackcloud P. Gilmore E. S. VanBuskirk J. Ritchlin C. T. (2018). Chronic social stress Ameliorates psoriasiform dermatitis through upregulation of the Hypothalamic-Pituitary-Adrenal axis. *Brain Behav. Immun.* 68 238–247. 10.1016/j.bbi.2017.10.022 29080684 PMC5767548

[B32] Woehlbier U. Hetz C. (2011). Modulating stress responses by the UPRosome: a matter of life and death. *Trends Biochem. Sci.* 36 329–337. 10.1016/j.tibs.2011.03.001 21482118

[B33] Wu F. Qiu J. Fan Y. Zhang Q. Cheng B. Wu Y. (2018). Apelin-13 attenuates ER stress-mediated neuronal apoptosis by activating Gαi/Gαq-CK2 signaling in ischemic stroke. *Exp. Neurol.* 302 136–144. 10.1016/j.expneurol.2018.01.006 29337146

[B34] Yan W. Frank C. L. Korth M. J. Sopher B. L. Novoa I. Ron D. (2002). Control of PERK eIF2alpha kinase activity by the endoplasmic reticulum stress-induced molecular chaperone P58IPK. *Proc. Natl. Acad. Sci. U.S.A.* 99 15920–15925. 10.1073/pnas.252341799 12446838 PMC138540

[B35] Yang J. W. Hu Z. P. (2015). Neuroprotective effects of atorvastatin against cerebral ischem/reperfusion injury through the inhibition of endoplasmic reticulum stress. *Neural. Regen. Res.* 10 1239–1244. 26487850 10.4103/1673-5374.162755 PMC4590235

[B36] Yi S. Shi W. Wang H. Ma C. Zhang X. Wang S. (2017). Endoplasmic reticulum stress PERK-ATF4-CHOP pathway is associated with hypothalamic neuronal injury in different durations of stress in rats. *Front. Neurosci.* 11:152. 10.3389/fnins.2017.00152 28392758 PMC5364325

[B37] Zhou Y. Lee J. Reno C. M. (2011). Regulation of glucose homeostasis through a XBP-1-FoxO1 interaction. *Nat. Med.* 17 356–365. 10.1038/nm.2293 21317886 PMC3897616

